# Differences in medical schools’ regional retention of physicians by school type and year of establishment: effect of new schools built under government policy

**DOI:** 10.1186/s12913-015-1240-2

**Published:** 2015-12-30

**Authors:** Satoru Kamitani, Fumiaki Nakamura, Mitsuko Itoh, Takehiro Sugiyama, Satoshi Toyokawa, Yasuki Kobayashi

**Affiliations:** Department of Public Health, Graduate School of Medicine, The University of Tokyo, 7-3-1 Hongo, Bunkyo-ku, Tokyo, 113-0033 Japan; Department of Pediatrics, The University of Tokyo Hospital, Tokyo, Japan; Department of Clinical Study and Informatics, Center for Clinical Sciences, National Center for Global Health and Medicine, Tokyo, Japan

**Keywords:** Physician shortage, Physician maldistribution, Establishing medical schools, Retention rates, Health policy

## Abstract

**Background:**

Physician maldistribution is an ongoing concern globally. The extent of medical schools retaining graduates within their geographical areas has rarely been explored in Japan or in other countries. This study aimed to investigate whether the proportion of medical school graduates practicing in the vicinity of medical school (retention rate) differs by the year of the school’s establishment and by the school’s funding source.

**Methods:**

This cross-sectional study used a set of databases on medical institutions and personnel. We analyzed a sample of 168,594 clinically active physicians practicing in institutions as of May 2014, who passed the National Medical Practitioners Examination between 1985 and 2013. We assessed the retention rate and the schools’ establishment period and funding source (pre-1970/post-1970, private/public), using a hierarchical regression model with random intercept unique to each medical school. We used the following factors as covariates: gender, physicians’ length of professional experience, and the geographical features of the medical schools.

**Results:**

The retention rate was widely distributed from 16.2 to 81.5 % (median: 48.4 %). Physicians who graduated from post-1970 medical schools were less likely to practice in the prefecture of their medical school location, relative to those who graduated from pre-1970 medical schools (adjusted odds ratio: 0.75; 95 % confidence interval: 0.62–0.90). Physicians who graduated from private medical schools were also less likely to practice in the prefecture of their medical school location, relative to those who graduated from public medical schools (adjusted odds ratio: 0.63; 95 % confidence interval: 0.51–0.77). In addition, the ability to retain graduates varied by school according to the school’s characteristics.

**Conclusions:**

There was a considerable difference between medical schools in retaining graduates locally. The study results may have significant implications for government policy to alleviate maldistribution of physicians in Japan.

**Electronic supplementary material:**

The online version of this article (doi:10.1186/s12913-015-1240-2) contains supplementary material, which is available to authorized users.

## Background

An adequate supply of healthcare personnel is critical for healthcare in any country [1, 2]. Regional shortages of physicians are attributed not only to overall shortages, but also to a maldistribution geographically and among specialities [3–9]. The characteristics of medical schools may play a role in influencing physicians’ choices regarding specialty and practice location.

In Japan, 34 new medical schools were established during the 1970s and early 1980s, with the intention of building at least one medical school in each prefecture to tackle the issue of physician shortage and maldistribution. In consequence, the number of physicians in Japan increased and the target number of 150 physicians per 100,000 populations was achieved in 1984 [10]. Since then, physicians have been steadily supplied, but with different increase rates by area; the growth rate of medical doctors from 1986 to 2010 by prefecture was 145.5 % on average, and the rate varied geographically from 129.7 to 205.9 % [11]. However, as Additional file 1: Table S1 shows, even though the number of physicians increased in total, geographical maldistribution has persisted and still remains an issue [4, 6, 12–14]. Maldistribution among specialties is also a persistent problem [9]. A postgraduate residency training program implemented in 2004, which permitted medical graduates to choose their preferred residency setting through a national matching system, is considered to have accelerated the physician maldistribution [15, 16].

Several studies have examined the characteristics of physicians in relation to rural practice [17, 18]. However, no studies have been conducted to see whether medical schools have been successful in retaining graduates within their located prefectures in Japan. Previous studies used either graduates’ information from a single medical school, or the national physician survey, which did not include any information on medical schools. To the best of our knowledge, even globally, few studies have examined whether characteristics of medical schools were relevant to retention of physicians in local communities. In this study, we explored whether physicians remained in the prefectures of their graduated schools, and the characteristics of medical schools that affect geographical retention, using nationwide data.

## Methods

### Design and database

We conducted a cross-sectional study using the Medical Database, established by a private market research company in Japan (Nihon Ultmarc Incorporated, URL: http://www.ultmarc.co.jp/). This database contains information gathered from medical institutions and healthcare providers in Japan. The database was originally compiled by medical representatives of pharmaceutical companies, and organized by Ultmarc for use in sales and marketing by pharmaceutical and medical device companies. Medical representatives acquire information on physicians who practice in medical institutions from the institutions’ websites, and augment this information by direct inquiry and interviews with physicians. Each physician’s name and year of registration are confirmed through the website of the Ministry of Health, Labour and Welfare of Japan, which also discloses medical certification information for each physician [19]. This database is updated regularly; the version used in the study is that of May 2014. As of that date, at least 82.0 % of medical licensees were included in the database. We obtained the data in an anonymous format.

### Inclusion and exclusion criteria

We included in our study sample physicians who had passed the National Medical Practitioners Examination between 1985 and 2013. In Japan, all medical school graduates must pass the National Medical Practitioners Examination to qualify as practicing physicians. We selected 1985 as the first year of the inclusion period because almost all of the newest medical schools began to supply graduates following this year. We excluded physicians with less than 2 years’ experience because they were junior residents and had not yet determined their specialties. We also excluded physicians whose schools were not named in the database, and those who worked in nonclinical fields, such as biomedical research or public health services. We also excluded physicians who graduated from Jichi Medical University, National Defense Medical College, and the University of Occupational and Environmental Health, because these medical schools impose practicing locations and/or specialties upon graduates as a condition of their scholarships.

### Outcome variables and covariates

We defined the primary outcome as the proportion of those physicians who practiced at clinical institutions in the prefecture of their medical school to the total number of physicians who had graduated from each school during the study period. Prefectures are municipal provinces in Japan, of which there are 47 throughout the country. The secondary outcome measure was the proportion of graduates who practiced at clinical institutions either in the prefecture of their medical school or in neighboring prefectures. These proportions are referred to as “retention rates” hereafter.

With respect to covariates, we adjusted for sex, number of years’ experience as a physician, year in which the medical school was established, whether the school was public or private, and the school’s location (whether it was located in a densely or sparsely populated city), to estimate the effects of individual and institutional factors. We categorized the number of years’ experience as a physician, in consideration of the usual career path of physicians, as follows: 3–5 years, 6–10 years, 11–20 years, and more than 20 years. We divided all Japanese medical schools into two groups: established pre-1970 or post-1970. Most medical schools established post-1970 were intended to solve physician shortages and maldistribution. Additional file 2: Table S2 lists the 46 medical schools established pre-1970, and the 31 schools established post-1970, excluding the three schools mentioned above. Medical schools were also divided according to funding source (public or private). There were 13 private schools in the pre-1970 group, and 14 private schools in the post-1970 group. In Japan, the number of students enrolled in each medical school is about 100 per year irrespective of the funding source, whereas the total tuition required is approximately 3.5 million JPY (≒US $29,160, as of October 22, 2015) for public medical schools and 20–45 million JPY (≒US $166,160–333,300, as of October 22, 2015) for private schools. The tuition burden differences between public and private schools were related to the practice settings and incomes of physicians in the United States [20]; therefore we suspected that it would be an influential factor in their choice of school and employment location subsequent to graduation in Japan. To assess the effect of medical schools located in urban areas, considered to attract physicians, we adjusted for the location of medical schools in prefectures that included cities with populations of more than 700,000.

### Statistical analysis

We performed descriptive analyses of the demographic characteristics for each category. We used chi-squared tests to compare categorical variables. To perform outcome analyses, we calculated the retention rates by medical schools according to their year of establishment (pre-1970 or post-1970) and their funding source (public or private). In the multivariate analysis, we used a random-intercept hierarchical regression model with intercepts unique to each medical school, to assess the remaining school characteristics after adjusting for year of establishment, funding (public or private), and location. We did additional analyses of the secondary outcome in a similar manner. Neighboring prefectures were defined as prefectures that share borders on land.

All statistical analyses were performed using STATA version 13.0 (StataCorp LP, College Station, TX, USA). All tests were two-tailed, and *p* < 0.05 was considered statistically significant.

### Ethical approval

On the basis of Ethical Guidelines for Medical and Research Involving Human Subjects in Japan, the Institutional Review Board of The University of Tokyo approved this research and determined that individual informed consent was not required (No. 10421).

## Results

**Fig. 1:**
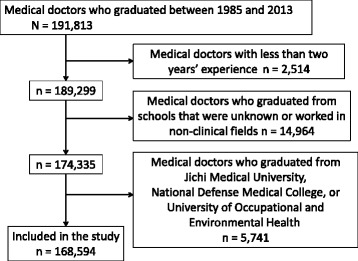
Flow chart for inclusion criteria

We analyzed a sample of 168,594 physicians. Figure 1 depicts the flowchart for sample selection. Table 1 lists subjects’ demographic characteristics, categorized according to the school’s funding mechanism and year of establishment. The numbers of years’ experience were similar across categories. Physicians who graduated from private medical schools were more likely to be in private practice relative to public medical school graduates. Private medical schools, regardless of the year of establishment, tended to be located in prefectures with densely populated cities, unlike public medical schools established post-1970.

**Table 1:** Demographics and characteristics of subjects categorized according to school’s year of establishment and funding sources^a^

Year of establishment	Pre-1970		Post-1970		
Funding source (n)	Public (*n* = 73,500)	Private (*n* = 29,758)	Public (*n* = 33,943)	Private (*n* = 31,393)	*p* value^*^
Sex (male)	81.0	74.2	74.9	74.5	<0.001
Experience					
3–5 years	7.3	7.0	7.9	7.5	<0.001
6–10 years	15.4	15.5	16.2	15.2	
11–20 years	38.5	39.3	39.5	39.9	
≥21 years	38.8	38.2	36.4	37.4	
Physicians in private practice	14.5	24.3	15.7	31.9	<0.001
Location (prefecture with densely populated cities^b^)	56.5	93.7	13.7	78.8	<0.001

^a^Data are reported as proportions of participants

^b^Medical schools located within prefectures that include municipalities of more than 700,000 residents

^*^ *p* values for chi-square tests for four categories

**Fig. 2:**
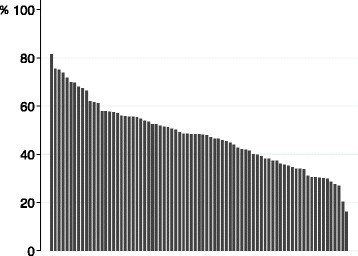
Distribution of retention rates for graduates practicing in the prefecture of their medical school. We defined the retention rate as the proportion of those physicians who practiced at clinical institutions in the prefecture of their medical school to the total number of physicians who had graduated from each school during the study period. Retention rates are sorted according to rank

**Fig. 3:**
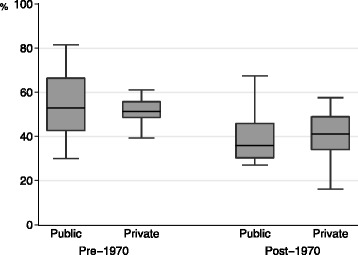
Retention rates for physicians practicing in the prefecture of their medical school. Retention rates are categorized according to the year of the school’s establishment (pre-1970 or post-1970) and funding mechanism (public or private). Horizontal lines in each box plot represent lower adjacent, 25th percentile, median, 25th percentile, and upper adjacent positions from the bottom

Figure 2 shows the retention rates for graduates practicing in the prefecture of their medical school location (the primary outcome). This rate ranged widely, from 16.2 to 81.5 %, with a median of 48.4 %. Figure 3 shows the retention rates for medical schools categorized according to year of establishment and funding mechanism. The range of retention rates varied according to category. Pre-1970 public medical schools demonstrated the largest range of proportions (from 30.1 to 81.5 %), while pre-1970 private medical schools showed the smallest range of proportions (from 38.0 to 61.0 %). The median proportions of pre-1970 medical schools were higher than those of the post-1970 medical schools (53.5, 51.3, 37.4, and 40.0 % for pre-1970 public, pre-1970 private, post-1970 public, and post-1970 private medical schools, respectively).

Table 2 lists the adjusted odds ratios (adjusted ORs) and 95 % confidence intervals (CIs) for factors influencing the retention of practicing graduates in the prefecture of their medical school location. Physicians who graduated from post-1970 medical schools were less likely to practice in the prefecture of their medical school relative to those who graduated from pre-1970 medical schools (adjusted OR: 0.75; 95 % CI: 0.62–0.90). Physicians who graduated from private medical schools were also less likely to practice in the prefecture of their medical school relative to those who graduated from public medical schools (adjusted OR: 0.63; 95 % CI: 0.51–0.77). Those who graduated from medical schools located in prefectures containing densely populated cities tended to practice in the prefecture of their medical school. Being male was significantly related to retention. Physicians tended to leave the prefectures gradually as they gained experience. After adjusting for year of establishment and location, the remaining variance was random; therefore, the ability to retain graduates varied according to school characteristics, in addition to the year of establishment, funding mechanism, and location.

**Table 2:** Effects of physicians’ characteristics on practicing in the prefecture of their medical schools

Fixed part	Adjusted odds ratio	95 % CI	*p* value
Year of establishment (post-1970)	0.75	0.62–0.90	0.002
School type (private)	0.63	0.51–0.77	<0.001
Location (prefecture with densely populated cities^a^)	2.34	1.92–2.85	<0.001
Sex (male)	1.07	1.05–1.10	<0.001
Experience			
3–5 years	1.24	1.19–1.29	<0.001
6–10 years	1.12	1.08–1.15	<0.001
11–20 years	1.05	1.03–1.08	<0.001
≥21 years	Reference	-	-
Random part	Standard deviation	95 % CI	*p* value
Medical school	0.37	0.32–0.44	<0.001

*CI* confidence interval

^a^Medical schools located within prefectures that include municipalities of more than 700,000

**Fig. 4:**
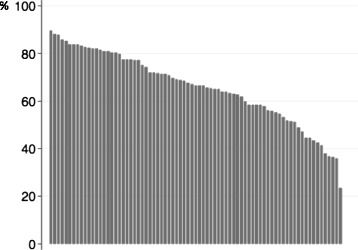
Distribution of retention rates in the additional analyses. Retention rates are the proportion of medical school graduates practicing in the prefecture of the medical school or neighboring prefectures. Retention rates are sorted according to rank

**Fig. 5:**
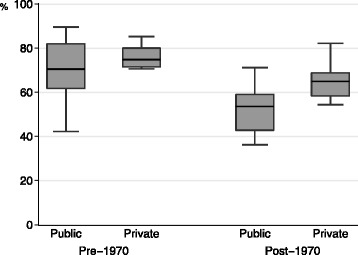
Retention rates for physicians in the additional analyses. Retention rates are the proportion of medical school graduates practicing in the prefecture of their medical school or neighboring prefectures. Retention rates are categorized according to year of the school’s establishment (pre-1970 or post-1970) and funding mechanism (public or private). Horizontal lines in each box plot represent lower adjacent, 25th percentile, median, 25th percentile, and upper adjacent positions from the bottom

Additional analyses of the secondary outcome, in which the definition of retention was broadened from practicing within the prefecture of the medical school to practicing within the prefecture of the medical school or neighboring prefectures, showed almost identical trends to those observed in the primary outcome (Figs. 4 and 5). However, physicians who graduated from private medical schools did not show a statistically significant tendency to practice beyond the borders of neighboring prefectures relative to those who graduated from public schools (adjusted OR: 0.85; 95 % CI: 0.66–1.08; Table 3). In contrast, physicians who graduated from post-1970 schools were more likely to practice beyond the borders of neighboring prefectures relative to those who graduated from pre-1970 schools (adjusted OR: 0.63; 95 % CI: 0.51 to 0.79; Table 3).

**Table 3:** Effects of physicians’ characteristics on practicing in the same or neighboring prefectures of their medical schools

Fixed part	Adjusted odds ratio	95 % CI	*p* value
Year of establishment (post-1970)	0.63	0.51–0.79	<0.001
School type (private)	0.85	0.66–1.08	0.178
Location (prefecture with densely populated cities^a^)	2.54	2.02–3.20	<0.001
Sex (male)	1.05	1.03–1.08	<0.001
Experience			
3–5 years	1.07	1.02–1.11	0.004
6–10 years	0.95	0.92–0.98	0.001
11–20 years	0.99	0.96–1.01	0.320
≥21 years	Reference	-	-
Random part	Standard deviation	95 % C.I.	*p* value
Medical school	0.43	0.37–0.51	<0.001

*CI* confidence interval

^a^Medical schools located within prefectures that include municipalities of more than 700,000 residents

## Discussion

The results of this study showed that 48 % of medical school graduates practiced in the prefecture in which the school was located; however, the school features influencing the retention of graduates varied considerably. Regardless of whether they were publicly or privately funded, the rates of retention were lower in medical schools established post-1970 and in those located in prefectures containing sparsely populated cities, relative to those established prior to 1970 and located in prefectures containing densely populated cities. In addition, aside from year of establishment and location, some characteristics of medical schools were associated with the proportion of graduates retained in the region.

This survey depicts the results of governmental policy to alleviate physician maldistribution by establishing at least one medical school in each prefecture. We found that post-1970 medical schools, which were established to reduce the shortage of physicians, were less likely to retain physicians in the prefecture of their medical school, relative to pre-1970 medical schools. The difference in retention between these two groups of medical schools may stem from an institutional culture that promotes a sense of solidarity derived from each school’s own traditions and history, as well as the degree of strength of the network between medical schools and local hospitals. In addition, schools located in densely populated districts exerted a positive effect on retention of graduates. Physicians may have preferred to practice in densely populated areas while attending medical school, as schools in these areas are believed to provide abundant hospital resources and opportunities for young physicians to experience various cases; furthermore, preference may have been based on factors that were unrelated to work [5, 21, 22]. Based on our findings and on previous US studies that indicated that publicly funded and locally situated medical schools tended to provide physicians in rural areas, the strategy to establish new medical schools in particular locations may not be the most effective way to remedy physician shortages in certain prefectures or regions, considering the vast expense and extensive human resources required, without bearing fruit in increasing retention [23, 24].

Similar to findings of previous studies in which public schools possessed more features through which to retain graduates, our study showed that physicians who graduated from public schools were more likely to remain in and around the prefecture of the school attended relative to those who graduated from private schools [22, 25]. Graduates from private schools may have been less resistant to leaving the local area subsequent to becoming physicians, as they had paid higher tuition fees. Another possibility is that insufficient education concerning regional healthcare is offered in private schools, and graduates may therefore fail to develop a desire to contribute to the local community. Although physicians have the right to select their location of employment and the field in which they wish to specialize, medical schools are responsible for contributing to the local community by providing an adequate number of physicians [26]. This is also true of private medical schools, as they are funded by the government to some extent.

Principal policy designed to reduce the shortage of physicians involves increasing the number of physicians per population [3, 4]. However, simply increasing the number of physicians has not addressed the issue of maldistribution; a location theory proposed by Newhouse [27], in which physicians spread to local areas once urban areas are saturated, does not reflect the distribution of physicians in the United States or Japan [4, 8, 28]. The Japanese government is currently implementing policies designed to increase the number of physicians. Increased recruitment of students who will remain in the local area has been approved for some medical schools, leading to the introduction of selective admission, via which a quota of local applicants is established, along with scholarships stipulating that graduates remain in the area surrounding the school. As selective admission has been considered an effective means of recruiting and retaining physicians in rural and underserved areas for some time, and financial incentives are one of the few evidence-based health policy interventions shown to retain physicians in local areas, these policies may be promising [17, 29–33]. We believe that comprehensive strategies to retain physicians in shortage areas, such as increasing medical school class sizes, introducing selective admission, organizing scholarships, offering intensive locally oriented programs, and expanding general practice and/or primary care medicine, would work [34–36].

Our study was subject to several limitations. First, the database used does not contain the details of all physicians in Japan. However, we confirmed that 82.0 % of medical licensees whose details were included in the database during the study period were accounted for. Physicians who work abroad or work in nonclinical fields subsequent to graduation from medical school in Japan are very limited. Second, with respect to information concerning the physicians’ schools examined in the study, misclassification by physicians or medical representatives, who collected the information, may have occurred. Therefore, we calculated the capture rate for each medical school to ensure that abnormally reported values did not exist; these rates ranged from 66.6 to 85.2 %, with a median of 78.4 % for the period between 1994 and 2011. Data regarding medical licensees for this period could be confirmed via reference to the data disclosed by the Ministry of Health, Labour and Welfare. Third, additional factors that we were unable to assess, such as hometown location and the economical background of medical students, may have affected the physicians’ choice of employment location. Fourth, we did not evaluate the distribution of physicians within prefectures in our analyses, and the evaluation of each prefecture as a unit may not have reflected the maldistribution of physicians between urban and rural areas. However, we used “prefecture” as the unit of outcome in order to focus on the nationwide distribution of physicians subsequent to graduation. Therefore, to evaluate the supply of manpower to areas experiencing a shortage of physicians, further detailed studies using smaller geographical units as outcomes are required.

## Conclusions

Medical schools’ ability to retain their practicing graduates in their own prefecture and the surrounding area differed considerably. In addition, medical schools established post-1970 were less likely to retain practicing graduates in the local region, relative to pre-1970 medical schools. The study results may have significant implications for government policy to alleviate maldistribution of physicians in Japan.

## Additional files

Additional file 1: Table S1. Number of physician per 100,000 population by prefecture in 2012. (DOCX 17 kb) Additional file 2: Table S2. Numbers of medical schools and student admissions in Japan between 1950 and 2010. (DOCX 17 kb)

## Abbreviations

OR: Odds ratio
CI: Confidence interval
