# Supplementary material for: Differences in medical schools’ regional retention of physicians by school type and year of establishment: effect of new schools built under government policy
Source: BMC Health Serv Res. 2015 Dec 30;15:581. doi: 10.1186/s12913-015-1240-2 (PMC4696324; doi:10.1186/s12913-015-1240-2)
Supplement: Additional file 1: Table S1. — Number of physician per 100,000 population by prefecture in 2012. (DOCX 17 kb) [file 12913_2015_1240_MOESM1_ESM.docx]

**Additional Table 1. Number of physician per 100,000 population by prefecture in 2012 ^a^**

| Prefecture | Number of physician per population ^a^ | Prefecture | Number of physician per population ^a^ | Prefecture | Number of physician per population ^a^ | Prefecture | Number of physician per population ^a^ |
| --- | --- | --- | --- | --- | --- | --- | --- |
| Average | 227 | Chiba | 173 | Mie | 197 | Tokushima | 296 |
| Hokkaido | 225 | Tokyo | 296 | Shiga | 205 | Kagawa | 260 |
| Aomori | 185 | Kanagawa | 194 | Kyoto | 297 | Ehime | 244 |
| Iwate | 190 | Niigata | 182 | Osaka | 257 | Kochi | 284 |
| Miyagi | 218 | Toyama | 233 | Hyogo | 227 | Fukuoka | 283 |
| Akita | 208 | Ishikawa | 264 | Nara | 218 | Saga | 250 |
| Yamagata | 210 | Fukui | 236 | Wakayama | 269 | Nagasaki | 276 |
| Fukushima | 179 | Yamanashi | 216 | Tottori | 280 | Kumamoto | 266 |
| Ibaragi | 167 | Nagano | 211 | Shimane | 262 | Oita | 257 |
| Tochigi | 205 | Gifu | 195 | Okayama | 277 | Miyazaki | 228 |
| Gunma | 215 | Shizuoka | 187 | Hiroshima | 246 | Kagoshima | 241 |
| Saitama | 148 | Aichi | 198 | Yamaguchi | 241 | Okinawa | 233 |

^a^ Number of physicians who practice in the clinical institutions per 100,000 population in each prefecture.

Source: Ministry of Health, Labour and Welfare
