# Supplementary material for: Differences in medical schools’ regional retention of physicians by school type and year of establishment: effect of new schools built under government policy
Source: BMC Health Serv Res. 2015 Dec 30;15:581. doi: 10.1186/s12913-015-1240-2 (PMC4696324; doi:10.1186/s12913-015-1240-2)
Supplement: Additional file 2: Table S2. — Numbers of medical schools and student admissions in Japan between 1950 and 2010. (DOCX 17 kb) [file 12913_2015_1240_MOESM2_ESM.docx]

**Additional Table 2. Numbers of medical schools and student admissions in Japan between 1950 and 2010**

| Year | | 1950 | 1960 | 1970 | 1980 | 1990 | 2000 | 2010 |
| --- | --- | --- | --- | --- | --- | --- | --- | --- |
| Public medical school ^a^ | Schools | 33 | 33 | 34 | 50 | 51 | 51 | 51 |
|  | Admissions | 2,020 | 2,040 | 2,980 | 5,220 | 4,825 | 4,810 | 5,690 |
| Private medical school ^b^ | Schools | 13 | 13 | 16 | 29 | 29 | 29 | 29 |
|  | Admissions | 880 | 800 | 1,400 | 3,040 | 2,925 | 2,885 | 3,241 |
| Total | Schools | 46 | 46 | 50 | 79 | 80 | 80 | 80 |
|  | Admissions | 2,900 | 2,840 | 4,380 | 8,260 | 7,750 | 7,695 | 8,931 |

^a^ National Defense Medical College is included in this category.

^b^ Jichi Medical University and University of Occupational and Environmental Health are included in this category. Because these medical schools impose practicing locations and/or specialties upon graduates as a condition of their scholarships, we did not include the study.

Source: Ministry of Education, Culture, Sports, Science and Technology
